# Feasibility and efficacy of a real-time smoking intervention using wearable technology

**DOI:** 10.1371/journal.pdig.0001086

**Published:** 2025-11-10

**Authors:** Krysten W. Bold, Luis M. Mestre, Kathleen A. Garrison, Ralitza Gueorguieva, Stephanie S. O’Malley, Lisa M. Fucito

**Affiliations:** 1 Department of Psychiatry, Yale School of Medicine, New Haven, Connecticut, United States of America; 2 Yale Cancer Center, New Haven, Connecticut, United States of America; 3 Department of Biostatistics, Yale School of Public Health, New Haven, Connecticut, United States of America; University of Bradford, UNITED KINGDOM OF GREAT BRITAIN AND NORTHERN IRELAND

## Abstract

Wearable technology can use gesture detection to identify smoking behavior and provide real-time feedback. Receiving notifications when smoking occurs may help increase awareness of smoking behavior to help promote change. The current study sought to examine the feasibility and preliminary efficacy of using a smartband for real-time smoking feedback as an adjunct to standard tobacco treatment in an outpatient hospital setting. We enrolled 38 adults (age M = 57.4, SD = 8.5, 63% female, race/ethnicity: 16% Hispanic, 68% White, 24% Black, 5% Multiracial) who smoked cigarettes daily (M = 17.2, SD = 10.9 cigarettes per day). All received standard tobacco treatment and participants were randomized to a control group (n = 20) or experimental group (receiving real-time smoking notifications from a smartband, n = 18) for 8 weeks. Participants wore the smartband on average for 45.6 (SD = 17.0) days out of the 56 days of treatment and 83.3% said they would recommend the smartband to others to help them quit smoking, indicating high adherence and satisfaction. Measures of smoking behavior favored the experimental group, although differences were not statistically significant. Rates of biochemically confirmed 7-day point-prevalence abstinence were 11% and 5% for the experimental and control groups, respectively. Those in the experimental group reported more percent days smoke-free (M = 12.4%, SD = 27.2% vs. control M = 6.9%, SD = 14.6%, cohen’s d = .26) and had larger reductions in cigarettes smoked per day (CPD) (mean change in CPD = 10.2, SD = 12.2 vs. control mean change in CPD = 7.7, SD = 6.5, cohen’s d = .26) during treatment. Findings support the feasibility of using smartband technology for smoking monitoring with adults from an outpatient hospital setting and show promise for improving cessation outcomes above and beyond standard tobacco treatment. Additional large-scale clinical trials are needed.

## Author summary

Quitting smoking is challenging and innovative treatments are needed to improve quitting success. Smoking is highly automatic, and people often smoke without thinking about it. We tested the use of wearable smartband technology that can monitor smoking in real time using sensors in the smartband. This technology can provide additional support by notifying people when they are smoking to help them become more aware of their smoking triggers and provide opportunities to try alternative coping strategies. We enrolled patients who were receiving tobacco treatment and tested whether adding a smartband that provided real-time smoking monitoring helped with quitting smoking. We discovered that people enjoyed wearing the smartband, found it helpful for reducing their smoking, and had more smoke-free days during treatment. These findings provide new information that smartbands may be a useful tool to help people quit smoking.

## 1. Introduction

Cigarette smoking remains the leading cause of preventable death in the United States [1], and over 37 million adults in the United States currently smoke cigarettes [2]. Although many adults who smoke make deliberate quit attempts each year, most attempts are unsuccessful [3–6] and utilization of evidence-based smoking treatments is low [7], thus more effective and scalable treatments are critically needed to enhance success in quitting. Quitting smoking is difficult in part because smoking is a highly automatic behavior [8–10] (e.g., lighting a cigarette without thinking about it), and automaticity of smoking is a central feature of nicotine dependence and a significant predictor of relapse [11]. Achieving and maintaining smoking abstinence requires, in part, being aware of smoking patterns and disrupting automatic behaviors to avoid smoking. Self-monitoring is an intervention strategy that is theorized to bring awareness to behavior and associated cues through event recording at the time that smoking occurs, thereby increasing perceived behavioral control and promoting behavior change, according to social-cognitive theories of behavior [12–15]. Evidence in support of this theory shows that self-monitoring is effective at reducing smoking [16–18]. However, problems with traditional self-initiated event monitoring (e.g., manually recording each smoked cigarette) include low adherence due to reasons such as fatigue, forgetting to record, and lack of real-time awareness of the behavior [19–21].

Wearable technology such as smartbands or smartwatches can provide an innovative solution to address cigarette smoking by providing automatic, continuous, and timely smoking assessment. These wearable devices are worn on the wrist and contain embedded geospatial sensors that use machine learning algorithms to identify the hand-to-mouth gestures associated with smoking a cigarette and notify the individual in real-time when smoking is detected, thereby bringing awareness to the otherwise automatic behavior of smoking. Several smartbands/watches have been initially validated to detect cigarette smoking in laboratory [22] and real-world settings [23–25], including using proprietary sensors [26,27] or off-the-shelf smartbands/watches [24,25,28]. Using smartbands to monitor and detect smoking may be advantageous due to their availability, ease of use, popularity, and potential for dissemination [29]. Preliminary research with the smartband used in the current study demonstrated high accuracy in detecting cigarette smoking (80–100% accuracy) with few false alarms (2–5%) indicating the smartband can reliably detect smoking and can differentiate smoking from other repeated hand gestures [28,30–32]. A pilot randomized controlled trial testing this smartband monitoring and notification system found a greater reduction in the number of cigarettes smoked per day after 30-days of wearing the study smartband compared to a wait-list control [31].

The current study built on this prior work by examining the feasibilty and preliminary efficacy of using a smartband to enhance quitting success among adults engaged in outpatient tobacco treatment. All participants received standard outpatient tobacco treatment as usual that included individual counseling and smoking cessation pharmacotherapy, and participants were randomized to receive either an active smartband for real-time smoking monitoring and notification (experimental group), or a control smartband (i.e., the same smartband but the smoking sensor was not active) for 8 weeks of treatment. The study aims were to: 1) evaluate the feasibility, acceptability, and helpfulness of the real-time smartband smoking intervention, and 2) assess the preliminary efficacy of the real-time intervention as an adjunct to standard tobacco treatment.

## 2. Methods

**Fig 1:**
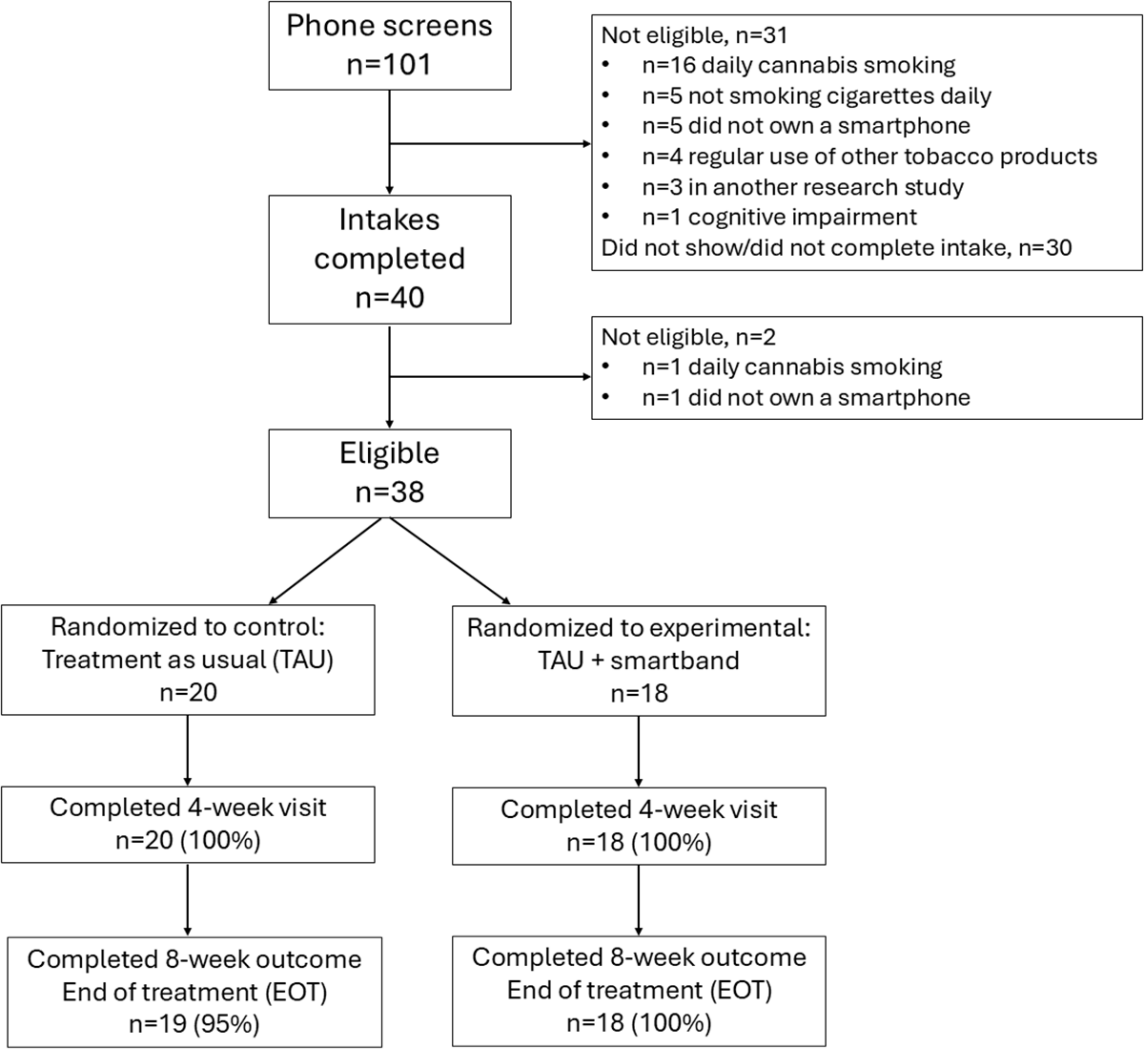
Consort diagram. Figure displays flow of participants from recruitment through randomization and retention.

All study procedures were approved by the Yale University IRB. We recruited adults from two outpatient hospital clinics from 2020-2021 at Yale-New Haven Hospital in Connecticut, USA, that provide individual tobacco treatment to adults who smoke. New patients that were referred to the tobacco treatment clinics who arrived for an intake appointment to enroll in tobacco treatment were offered the opportunity to participate in the study via clinician referrals. Research staff contacted potential participants to determine initial eligibility, and those who were interested in participating in the research study to test the adjunct smartband treatment met with research staff to obtain written informed consent and complete questionnaires to confirm eligibility and enroll in the study (see Fig 1, consort diagram). All research study procedures were conducted remotely, and participants were compensated via pre-paid debit card in amounts of $20 for each research visit with an additional $20 study completion bonus for completing the week 8 visit and returning the smartband and charger via prepaid envelope.

### 2.1 Participants

Eligibility criteria included: (a) age 18 years or older, (b) smoking ≥ 1 cigarette daily, (c) seeking smoking cessation treatment at one of the Yale outpatient tobacco treatment programs, (d) able to read and write in English, (d) owning an Android or iOS smartphone compatible with the study smartband. Exclusion criteria included: (a) self-reported serious or unstable psychiatric/medical conditions (e.g., suicidal ideation, acute psychosis, dementia), (b) current use of other tobacco products assessed via self-report. We excluded participants who reported current use of other tobacco products (e.g., cigars, e-cigarettes) or regular use of smoked cannabis because the study smartband is only validated to detect and respond to cigarette smoking.

### 2.2 Procedures

All participants received standard outpatient tobacco treatment (treatment as usual, TAU) following clinical practice guidelines [3]. TAU included receiving up to 8 sessions of one-on-one counseling focusing on setting a quit date and discussing practical behavioral strategies to support smoking cessation including coping with craving, handling withdrawal, and relapse prevention. Patients were encouraged to try smoking cessation pharmacotherapy and could obtain these medications over the counter or by prescription from their tobacco treatment provider.

All participants were shipped a study smartband (Somatix, Inc.) via priority mail (1–3 days) to wear for 8 weeks and were randomized to either the experimental or control smartband conditions. All participants completed a Zoom video-based visit to receive instructions about the smartband. Participants in both groups were instructed to wear the smartband during their normal waking hours and to take off and charge the smartband each night. Automated text messages were programmed to remind participants to charge their smartband prior to their usual bedtime and to put on the smartband first thing in the morning at their usual wake time. All participants were informed that the smartband might detect and notify them about their cigarette smoking, so those in the control group were not told that their band was inactive. Participants were informed that the goal of the smartband was to monitor cigarette smoking to provide feedback about smoking in the moment and to test if this was helpful for people for quitting smoking.

The control condition received a sham smartband that looked identical to the active smartband to control for demands associated with remembering to charge and wear the smartband daily. The only difference was that the control smartband was not connected to a smartphone app, so it was not activated for gesture detection and did not collect any data or send any notifications when smoking occurred.

Those in the experimental group with the active smartband downloaded the smoking detection app to their smartphone, paired the smartphone with the smartband via Bluetooth connection, and enabled notifications from the Somatix app. The active smartband provided continuous real-time monitoring and notifications when smoking was detected. Cigarette smoking was identified using hand gesture data from the embedded geospatial sensors in the smartband and classified as smoking by a proprietary machine-learning algorithm. Immediately upon detection of smoking (after ~2–3 puffs), the smartband vibrated once briefly, and a real-time notification was sent to the participant through the app on their phone. Participants were asked to respond to the real-time notification to confirm if they were smoking or not (i.e., the band has detected smoking, did you really smoke? Yes/No). Their response was used to update the smoking detection algorithm to enhance accurate detection of their smoking. Participants were also instructed how to manually report a smoking event in the app if the smartband did not detect it to further train the smoking algorithm to their smoking pattern. Participants could access the app at any time to view an ongoing count of cigarettes smoked by day, week, or month. The smartphone app did not provide any other intervention material to participants.

Eligible participants were randomized to: (1) TAU + experimental smartband or (2) TAU + control smartband. Randomization was allocated 1:1 and was stratified by average number of cigarettes smoked per day at baseline (<10 or ≥10 cigarettes per day) to balance groups on smoking intensity. Enrolled participants completed remote research assessments at week 0 (intake/randomization), week 4, and week 8. Procedures were initially piloted (n = 11 experimental, n = 9 control), and changes were made to the protocol including updating the smartband software and revising all procedures to be fully remote prior to launching the current study. The current study included n = 18 participants randomized into the experimental group and n = 20 into the control group (Table 1).

**Table 1:** Demographic and cigarette use characteristics of the sample overall and by treatment group.

	Experimental (N = 18)	Control (N = 20)	Overall (N = 38)
Demographic characteristics			
Age (M, SD)	57.2 (7.5)	57.6 (9.6)	57.4 (8.5)
Female (N, %)	10 (55.6%)	14 (70.0%)	24 (63.2%)
Race/Ethnicity^1^ (N, %)			
Hispanic	1 (5.6%)	5 (25.0%)	6 (15.8%)
White	11 (61.1%)	15 (75.0%)	26 (68.4%)
Black/African American	5 (27.8%)	4 (20.0%)	9 (23.7%)
Native American/Alaska Native	0 (0.0%)	0 (0.0%)	0 (0.0%)
Asian	0 (0.0%)	0 (0.0%)	0 (0.0%)
Native Hawaiian or Pacific Islander	0 (0.0%)	0 (0.0%)	0 (0.0%)
More than one race	1 (5.6%)	1 (5.0%)	2 (5.3%)
Unknown	0 (0.0%)	1 (5.0%)	1 (2.6%)
Employment status (N, %)			
Working fulltime	4 (22.2%)	7 (35.0%)	11 (28.9%)
Working parttime	2 (11.1%)	2 (10.0%)	4 (10.5%)
Retired	3 (16.7%)	6 (30.0%)	9 (23.7%)
Disabled	5 (27.8%)	3 (15.0%)	8 (21.1%)
Unemployed	4 (22.2%)	1 (5.0%)	5 (13.2%)
Homemaker	0 (0.0%)	0 (0.0%)	0 (0.0%)
Not reported	0 (0.0%)	1 (5.0%)	1 (2.6%)
Highest Education Completed (N, %)			
Less than high school	3 (16.7%)	2 (10.0%)	5 (13.2%)
High School or GED	6 (33.3%)	6 (30.0%)	12 (31.6%)
College 1–3 years	8 (44.4%)	6 (30.0%)	14 (36.8%)
College graduate	0 (0.0%)	3 (15.0%)	3 (7.9%)
Advanced or Associates degree	1 (5.6%)	2 (10.0%)	3 (7.9%)
Not reported	0 (0.0%)	1 (5.0%)	1 (2.6%)
Medicaid Insurance (N, %)	9 (50.0%)	9 (45.0%)	18 (47.4%)
Medical Comorbidity^1^, Any (N, %)	16 (88.9%)	20 (100.0%)	36 (94.7%)
Hypertension	8 (44.4%)	9 (45.0%)	17 (44.7%)
COPD/Emphysema	8 (44.4%)	7 (35.0%)	15 (39.5%)
Cancer	6 (33.3%)	5 (25.0%)	11 (28.9%)
Heart Disease	6 (33.3%)	5 (25.0%)	11 (28.9%)
Diabetes	8 (44.4%)	2 (10.0%)	10 (26.3%)
Obesity	5 (27.8%)	5 (25.0%)	10 (26.3%)
Stroke	0 (0.0%)	2 (10.0%)	2 (5.3%)
Cigarette use characteristics			
Cigarettes per day, CPD (M, SD)	18.6 (11.5)	16.0 (10.4)	17.2 (10.9)
Number of years smoked (M, SD)	38.7 (10.2)	37.8 (11.6)	38.2 (10.8)
Cigarette dependence^2^ (M, SD)	13.9 (3.7)	13.4 (3.0)	13.7 (3.4)
Quitting importance^3^ (M, SD)	9.3 (1.3)	9.4 (1.7)	9.5 (1.4)
Quitting confidence^3^ (M, SD)	7.2 (2.5)	7.2 (1.9)	7.1 (2.4)

Note: ^1^Response options are select all that apply, so values may add up to > 100%. ^2^Cigarette dependence measured by the 4-item PROMIS scale, range 4–20, with higher scores indicating greater dependence, corresponding to T-scores between 52.8-55.1 [33]. ^3^Quitting importance and confidence rated from 0 (not at all) to 10 (extremely).

### 2.3 Measures

#### 2.3.1 Baseline self-report measures.

Baseline measures were used to characterize the sample including demographic characteristics (e.g., age, gender, race [select all that apply], ethnicity [Hispanic yes/no], marital status, employment status, occupation), smoking history (e.g., cigarettes smoked per day, number of years smoked, nicotine dependence [33]), quitting motivation and confidence using the contemplation ladder, rated from 0 (not at all) to 10 (extremely) [34].

#### 2.3.2 Feasibility and acceptability outcomes.

Feasibility measures included objective measures of smartband adherence (i.e., the number of days wearing the smartband, number of hours per day wearing the smartband). Additionally, accuracy of smartband detection of smoking was evaluated as the percent of smoking notifications confirmed out of the total cigarettes detected. Acceptability measures included self-report ratings at the end of treatment (week 8) evaluating participant satisfaction, perceived helpfulness of the smartband, and ease of use of the smartband. Satisfaction was measured on a 5-point scale with items assessing satisfaction with the treatment received (1 = very dissatisfied, 2 = moderately dissatisfied, 3=neither satisfied nor dissatisfied, 4 = moderately satisfied, 5 = very satisfied), overall quality of the treatment received (1 = poor, 2 = fair, 3 = average, 4 = good, 5 = excellent), willingness to return to the program again and willingness to wear the smartband again (rated 1 = definitely not, 2 = probably not, 3 = maybe, 4 = probably yes, 5 = definitely yes), and 2 items asking 1) if they would recommend this program to others, and 2) if they would recommend the smartband to others to help them quit smoking (yes vs. no) (see Table 2). Participants rated helpfulness of the treatment components including counseling, medications, wearing the smartband to track smoking, and the program overall on a 5-point scale (1 = not at all helpful, 2 = slightly helpful, 3 = somewhat helpful, 4 = very helpful, 5 = extremely helpful). Participants rated ease of use of the smartband with several items including if the smartband was comfortable to wear, easy to use, they enjoyed wearing, interfered with daily activities or sleep, and difficult to remember to wear rated on a 5-point scale (1 = strongly disagree, 2 = disagree, 3=neither agree nor disagree, 4 = agree, 5 = strongly agree).

**Table 2:** Acceptability ratings of satisfaction, helpfulness, and ease of use of the smartband by group.

	Experimental Group N = 18		Control Group N = 20	
Intervention satisfaction	Mean (SD) or N(%)	N (%) who rated 4 or 5 on 5 point scale	Mean (SD) or N(%)	N (%) who rated 4 or 5 on 5 point scale
How satisfied are you with the treatment you received^1^	4.2 (1.2)	14 (77.8%)	4.1 (0.8)	13 (65.0%)
How would you rate the quality of the treatment you received^2^	4.2 (1.1)	14 (77.8%)	4.1 (0.8)	13 (65.0%)
Would you be willing to return to this program again to help you quit smoking^3^	4.5 (0.9)	15 (83.3%)	4.2 (0.9)	14 (70.0%)
Would you be willing to wear the smartband again to help you quit smoking^3^	4.2 (1.2)	14 (77.8%)	3.8 (1.2)	12 (60.0%)
Would you recommend the program to others to help quit smoking? Yes (vs. No)	17 (94.4%)	–	18 (90.0%)	–
Would you recommend the smartband to others to help quit smoking? Yes (vs. No)	15 (83.3%)	–	15 (75.0%)	–
Intervention helpfulness^4^	Mean (SD)	N (%) who rated 4 or 5 on 5 point scale (very/extremely helpful)	Mean (SD)	N (%) who rated 4 or 5 on 5 point scale (very/extremely helpful)
How helpful was one-on-one counseling	4.0 (1.2)	14 (77.8%)	3.6 (0.7)	8 (40.0%)
How helpful was the program overall	3.9 (1.1)	12 (66.7%)	3.6 (0.9)	10 (50.0%)
How helpful were medications	3.4 (1.3)	11 (61.1%)	3.1 (1.3)	7 (35.0%)
How helpful was wearing the smartband to track smoking	3.4 (1.6)	10 (55.5%)*	2.6 (1.1)	3 (15.0%)*
Smartband ease of use^5^	Mean (SD)	N (%) who rated 4 or 5 on 5 point scale (agree/strongly agree)	Mean (SD)	N (%) who rated 4 or 5 on 5 point scale (agree/strongly agree)
The smartband was easy to use	3.7 (1.3)	14 (77.8%)	4.1 (1.0)	15 (75.0%)
I did not have to change my daily routine in order to comply with wearing the smartband	3.9 (1.2)	12 (66.7%)	4.1 (0.9)	15 (75.0%)
I enjoyed wearing the smartband	3.4 (1.1)	10 (55.5%)	3.1 (1.1)	6 (30.0%)
I found it difficult to remember to wear the smartband each day	3.4 (1.1)	9 (50.0%)	3.2 (1.0)	7 (35.0%)
The smartband was comfortable to wear	3.3 (1.1)	8 (44.4%)	3.9 (1.0)	13 (65.0%)
Wearing the smartband interfered with my sleep	2.9 (1.2)*	6 (33.3%)	1.9 (1.0)*	1 (5.0%)
Wearing the smartband interfered with my daily activities	2.4 (1.0)	3 (16.7%)	2.2 (1.0)	1 (5.0%)

Note: *p < .05, t-test comparison of mean values or Fisher’s exact test comparison of frequencies between groups. ^1^Satisfied response options (1 = very dissatisfied, 2 = moderately dissatisfied, 3=neither satisfied nor dissatisfied, 4 = moderately satisfied, 5 = very satisfied); ^2^Treatment quality response options (1 = poor, 2 = fair, 3 = average, 4 = good, 5 = excellent); ^3^Willing to use again response options (1 = definitely not, 2 = probably not, 3 = maybe, 4 = probably yes, 5 = definitely yes); ^4^Helpfulness response options (1 = not at all helpful, 2 = slightly helpful, 3 = somewhat helpful, 4 = very helpful, 5 = extremely helpful); ^5^Ease of use response options (1 = strongly disagree, 2 = disagree, 3=neither agree nor disagree, 4 = agree, 5 = strongly agree)

#### 2.3.3 Smoking outcomes.

The timeline follow-back interview (TLFB) [35,36] was completed via Zoom or phone-based interview at each visit (baseline, week 4, week 8) to assess daily smoking. The primary smoking outcome was 7-day point-prevalence abstinence (PPA) at the end of treatment (week 8), measured via self-report on the TLFB. Those who reported smoking abstinence were shipped carbon monoxide (CO) devices via priority next-day mail and expired breath CO readings were done remotely via Zoom video visit. Smoking abstinence was confirmed with expired breath carbon monoxide (CO) levels ≤ 4ppm. Additional secondary smoking outcomes included percent days smoke-free during the 8 weeks of treatment (measured continuously with TLFB data) and differences in cigarettes smoked per day between baseline and end of treatment (week 8).

### 2.4 Data analysis

To assess feasibility, we used descriptive statistics 1) to characterize adherence using the smartband device based on self-report and objective adherence and 2) to summarize participant ratings of satisfaction and perceived usefulness of the intervention at the end of treatment. Fisher’s exact tests and independent samples t-tests were used to compare feasibility and acceptability measures between groups. To assess potential efficacy of the experimental vs. control conditions, we compared biochemically confirmed 7-day PPA at the end of treatment (i.e., reported abstinence from cigarettes during the last week of the study that was confirmed with CO ≤ 4ppm) to estimate effect sizes between groups. We used an intent to treat analysis, where all randomized participants were included in the primary outcome analysis examining biochemically-confirmed 7-day PPA. Missing PPA (1 participant in the control group) was coded as not abstinent. We also evaluated secondary continuous smoking outcomes comparing the percent days smoke-free during the 8 weeks of treatment and changes in cigarettes smoked per day from baseline to week 8 between groups. Both parametric and non-parametric tests (t-test, Wilcoxon Rank Sum Test) were conducted given the non-normal distribution of the outcomes to compare total percent days abstinent and changes in cigarettes per day between groups, and results were consistent. We conducted a repeated measures mixed effects model with time as a within-subject factor and group as a between-subject factor including an interaction of time x group to examine differences in cigarettes per day from baseline to week 8 between groups. This approach is flexible, uses all available data, and provides good statistical power with smaller sample sizes [37].

## 3. Results

### 3.1 Participants

We enrolled n = 38 adults (63% female, race/ethnicity: 16% Hispanic, 68% White, 24% Black, 5% Multiracial) who smoked cigarettes daily (M = 17.2, SD = 10.9 cigarettes per day). The average age of the sample was 57.4 years old, SD = 8.5, and most were middle aged (N = 27 45–64 years old) or older adults (N = 8 65–75 years old), who are a priority population for tobacco treatment research [38]. Descriptive characteristics of the sample overall and by treatment group (control n = 20, experimental n = 18) are presented in Table 1. There were no significant baseline differences between groups in any of the cigarette use measures or key demographic variables (e.g., age, gender, race, ethnicity, employment, education, medicaid insurance) (ps > .20). Overall, 47% of the sample had Medicaid insurance and almost all (94.7%) had a medical comorbidity, most commonly hypertension, COPD or emphysema, cancer, heart disease, or diabetes.

### 3.2 Feasibility and acceptability of the smartband smoking intervention

Intervention feasibility was measured with objective adherence data collected from the smartband for those in the experimental group with an active smartband. All participants in the experimental group (n = 18) wore the smartband and responded to real-time notifications confirming when they smoked. In total, 89.4% of cigarettes detected by the smartband were approved by the user as accurate detections (out of all detections), 10.5% of notifications were rejected as inaccurate detections, and < 1% were not responded to. Additionally, most participants (n = 17/18) manually added cigarettes if needed that were missed by the algorithm. Participants in the experimental group wore the smartband on average for 45.6 (SD = 17.0) days out of the 56 days of treatment (81%). The smartband had connectivity for M = 22.2 (SD = 4.8) hours per day and was worn for M = 8.5 hours per day on average (SD = 4.9). Additionally, self-reported rates of wearing the smartband daily were collected for both groups, and there was no difference in self-reported rates of wearing the smartband between the experimental and control group wearing the sham smartband (p > .70), indicating high rates of adherence.

Intervention acceptability was measured with participant self-report ratings of satisfaction, helpfulness, and ease of use of the experimental smartband that provided smoking monitoring and notifications (Table 2). Participants reported high ratings of satisfaction with the treatment and 77.8% (N = 14) were willing to wear the smartband again to quit smoking. Additionally, the majority indicated they would recommend the smartband to others (83.3%, N = 15) to help them quit smoking. Participants rated how helpful the treatment components were for quitting smoking and rated the smartband as somewhat to very helpful on average, similarly helpful to other standard treatment components like counseling and medication. For smartband ease of use, the highest reported ratings were that the smartband was easy to use, they enjoyed wearing it, and did not have to change daily routines to comply with wearing the smartband, although there was moderate agreement that it was difficult to remember to wear each day. On average, few people reported the smartband interfered with daily activities or sleep, although these ratings were slightly higher for those in the experimental vs. control group. Overall, participants in the experimental (vs. control) group reported similar ratings for intervention satisfaction and ease of use, and a significantly greater proportion of those in the experimental smartband group rated that it was very/extremely helpful wearing the smartband to track smoking (55.5%), compared to the control group (15%, Fisher’s exact test, p = .035), providing further confirmation of the acceptability of the active smartband (Table 2).

### 3.3 Changes in smoking outcomes to evaluate the smartband as an adjunct to standard treatment

**Fig 2:**
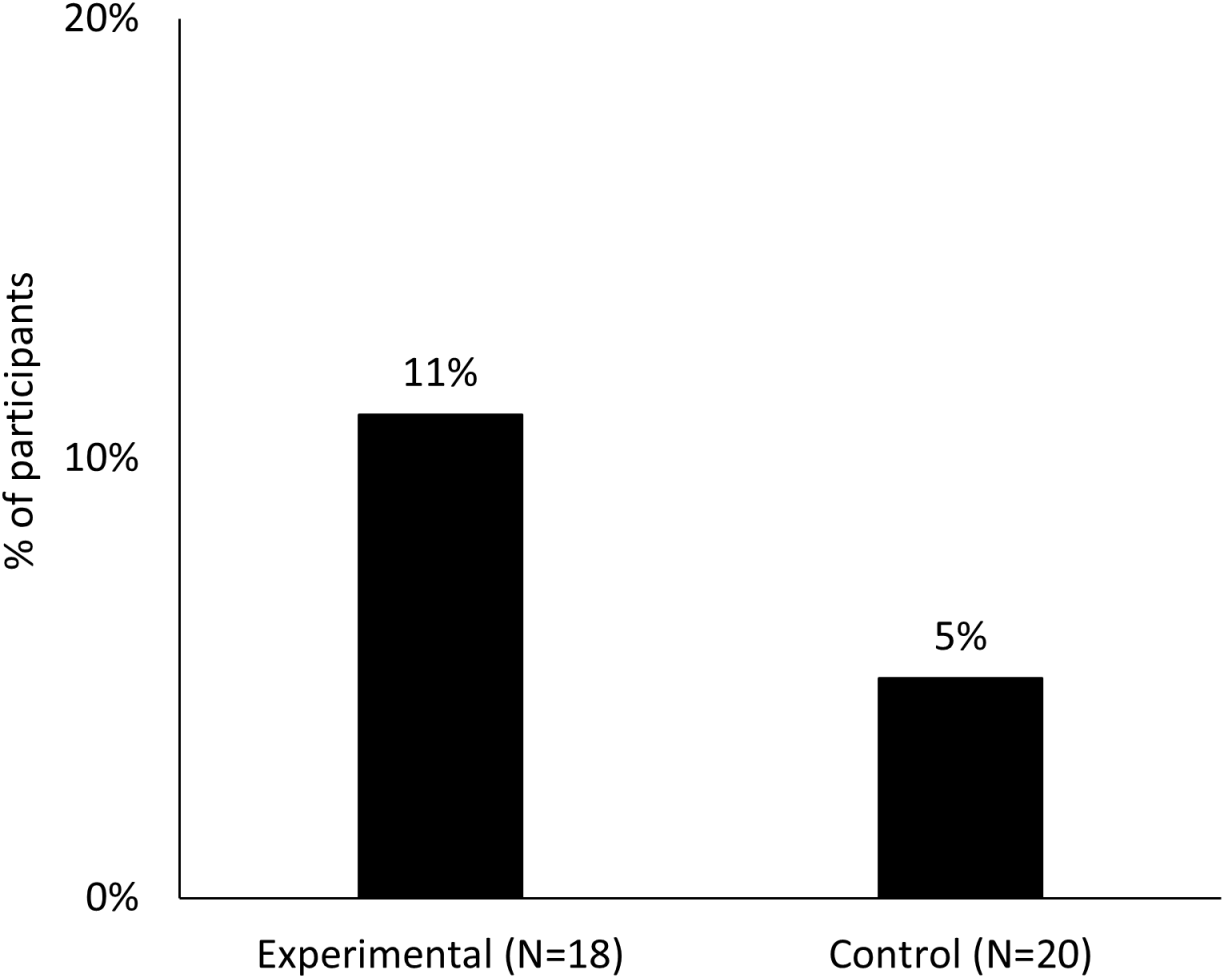
Rates of biochemically confirmed abstinence at end of treatment by condition. Figure displays the percent of participants achieving biochemically-confirmed 7 day point-prevalence abstinence at the end of treatment between the active smartband (experimental) and control conditions.

**Fig 3:**
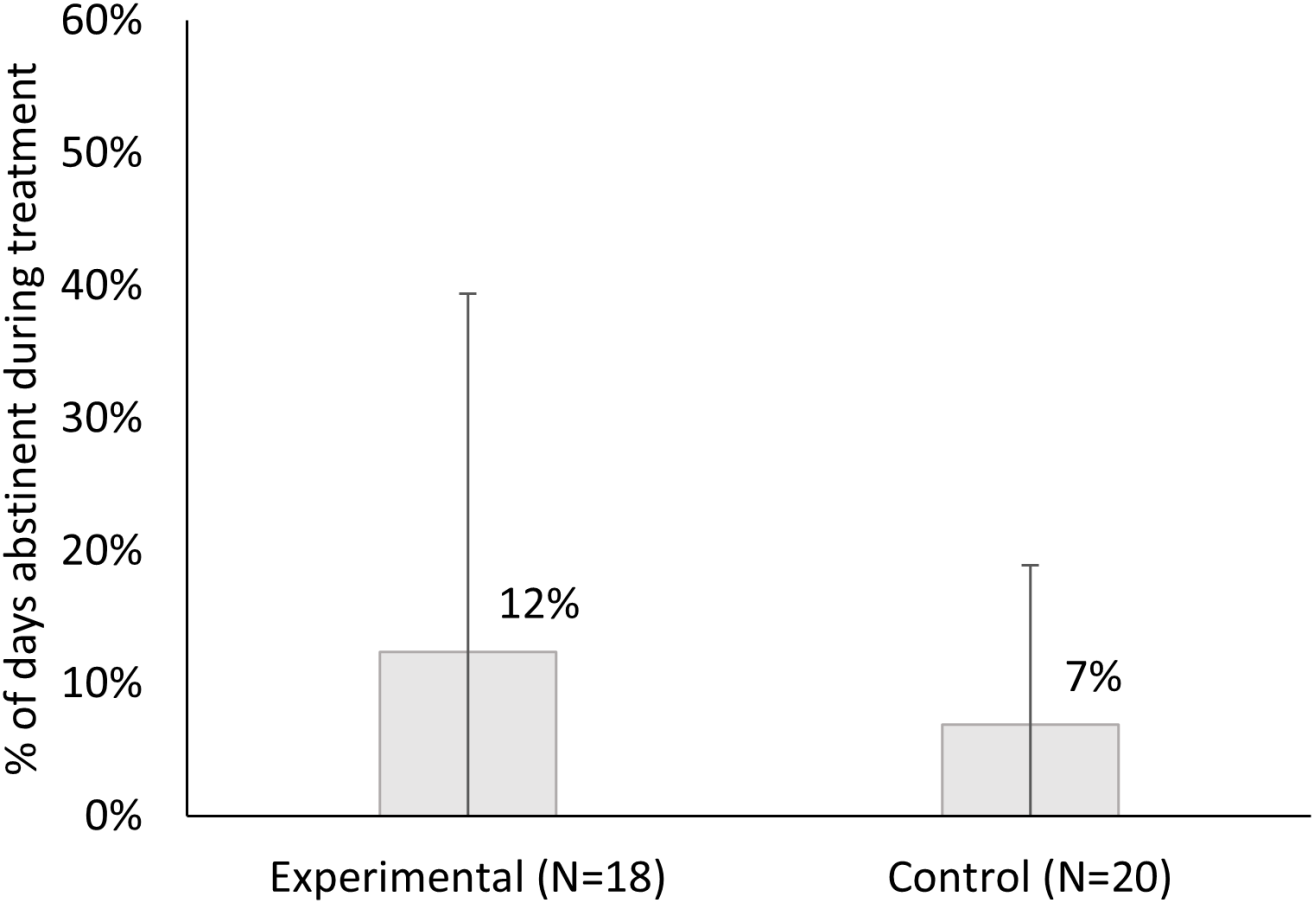
Percent days abstinent during treatment by condition. Figure displays the percent of days abstinent during treatment between the active smartband (experimental) and control conditions.

The primary outcome was biochemically confirmed 7-day PPA at the end of treatment (8 weeks), and abstinence rates were 11.1% (2/18) in the experimental group vs. 5.0% (1/20) in the control group, Fishers exact test p = 0.595 (Fig 2). Rates of self-reported (not confirmed) 7-day PPA were 2/18 in the experimental group and 3/20 in the control group. For secondary outcomes, the experimental group reported nearly double the percent days smoke-free during the 8-week treatment period (M = 12.4%, SD = 27.2%, range 0%-78% vs. control M = 6.9%, SD = 14.6%, range 0%-52%, Cohen’s d = .26), although this difference was not statistically significant (t(36)=0.78, p = .44; W = 169, p = .95) (Fig 3).

**Fig 4:**
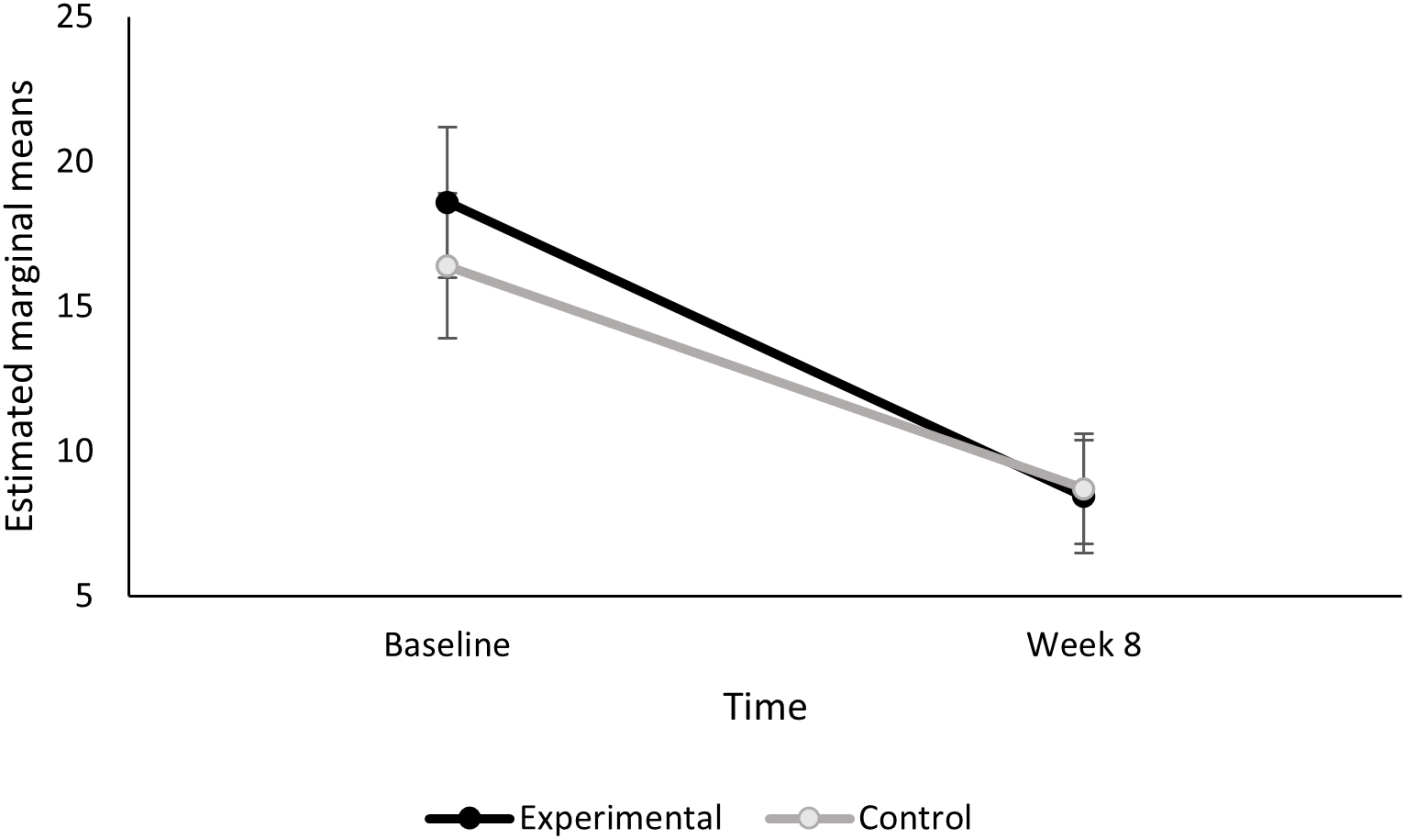
Estimated marginal means of cigarettes per day over time by condition. Figure displays the average cigarettes smoked per day between baseline and end of treatment between the active smartband (experimental, black line) and control (grey line) conditions.

For changes in cigarettes per day (CPD), there was a significant effect of time, F(1,35)=31.51, p < .001 where both groups had a significant reduction in CPD from baseline to end of treatment (week 8). The effect of time x condition on change in CPD was not statistically significant, F(1,35)=0.61, p = .44. Those in the experimental group had a numerically larger reduction in cigarettes smoked per day (CPD) from baseline to Week 8 (mean change in CPD = 10.2, SD = 12.1 vs. control mean change in CPD = 7.7, SD = 6.5, Cohen’s d = .26) (Fig 4).

## 4. Discussion

This randomized clinical trial evaluated the use of a wearable smartband for supporting smoking treatment among adults engaged in tobacco treatment in an outpatient hospital setting. There is potential for using wearable technology to support health behavior change such as quitting smoking, given the current availability and projected growth in the wearable market [39–41]. Notably, 41% of U.S. adults currently own a wearable device such as a smartwatch or wearable health tracker [42], suggesting that intervention programs that use this technology could have wide population reach. Study findings provide important new information about using a wearable smartband to support quitting smoking and provide considerations for future research.

This study is the first to test the use of wearable technology for tracking smoking among an adult sample from an outpatient hospital setting. We enrolled an older adult sample on average, typical of an outpatient hospital setting, and this population of adults has known difficulties successfully quitting smoking, given high rates of medical comorbidities, long histories of established cigarette smoking, and higher than average nicotine dependence scores [38,43,44]. Yet the findings showed it is feasible and acceptable among an older population to use smartband technology and to adhere to it, as demonstrated by objective data from the smartband showing it was worn for most days (81.4%) out of treatment. These rates of adherence are consistent with other studies using this smartband among younger participants recruited nationally [45]. Additionally, participants reported high ratings for treatment satisfaction, helpfulness, and ease of use of the smartband, and the majority reported they would be willing to use it again and would recommend it to others. However, 50% of the sample reported it was difficult to remember to wear the smartband each day, identifying an opportunity for improving intervention adherence. We programmed automated text messages twice daily to remind participants to charge their smartband prior to their usual bedtime and to put on the smartband first thing in the morning at their usual wake time, and future studies may want to consider additional reminders to support consistent use of the wearable technology to optimize adherence and enhance the potential clinical benefit. Importantly, larger studies are needed to establish what thresholds for adherence and use of wearable technology for tracking smoking are related to clinical improvement, as adherence across wearable studies is mixed and not well defined [23].

Additionally, this study shows potential efficacy for reducing smoking by using a smartband for tracking smoking as an adjunct to standard tobacco treatment among patients in an outpatient hospital setting. We observed differences in smoking outcomes between groups, although this pilot study was not sufficiently powered to detect statistically significant differences. Biochemically confirmed 7-day PPA rates at the end of treatment were higher in the experimental group (n = 2/18 11%) compared to the control group (n = 1/20 5%), and those in the experimental group reported more percent days smoke-free and had larger reductions in cigarettes smoked per day during treatment. These quit rates are comparable to other pilot smartband studies, including a single-arm study testing a real-time mindfulness intervention delivered alongside smartband-detected smoking events [45] and a randomized two-arm study that compared standard tobacco treatment (counseling and nicotine replacement therapy) alone to standard treatment plus a smartwatch to detect smoking among a sample of adults with HIV who smoked cigarettes daily [46]. Additional fully-powered studies are needed to test the efficacy of smartband technology for tracking smoking and providing real-time intervention opportunities for the general population of adults who smoke.

These findings should be considered in light of study strengths and limitations. Notably, this study tested a highly novel technology in a new patient population (adults from an outpatient hospital setting) using a randomized controlled design that compared the active smartband to a sham smartband control, which adds to the scientific rigor. This is the first study that tested a sham smartband in the control group, which is a stringent control that accounts for demand characteristics associated with remembering to charge and wear the band and having smoking behavior monitored continuously with the band, just without receiving notifications. We did not assess participant beliefs about whether their smartband was active or sham, although it is possible that participants were able to ascertain their study group. Additionally, there might be other factors related to treatment effects that could be considered in future studies like use of counseling and medication adherence. Furthermore, the study smartband has not been tested to differentiate cigarette smoking from other smoking behaviors, such as cannabis or other tobacco product use, so we recruited adults who smoked cigarettes only and excluded for other tobacco product use or regular cannabis use. No other tobacco product use was noted during the study and only infrequent cannabis use was reported. Further work is warranted to test the ability of the gesture detection algorithm to differentiate cigarette smoking from other similar gestures, such as cannabis or other tobacco product use, to expand the use of this novel technology. Lastly, the study was conducted among a sample of patients from tobacco treatment programs in Connecticut, so findings may not generalize to other populations. However, the research study was conducted fully remotely, providing further support for the feasibility of using this technology broadly.

Future research might consider additional opportunities to expand on the use of this wearable technology to provide real-time support for quitting. For example, studies have used smartband data to deliver real-time interventions at the time of smoking (e.g., mindfulness exercises) and in advance of predicted smoking events based on individual smoking patterns obtained from smartband data [45]. Thus, this technology could be used to build on studies that have tested the use of smartband technology alongside or as an adjunct to tobacco cessation pharmacotherapy like nicotine replacement therapy [46,47] by testing ways to optimize intervention delivery with the smartband such as encouraging real-time medication use or practicing coping skills in the moment to avoid smoking. Furthermore, as the wearable market continues to grow, additional studies are needed to evaluate the dissemination potential of this technology by testing the smoking tracking and monitoring app system with adults who already own smartwatches, as this smoking monitoring app is compatible across iOS and Android systems and works with existing smartwatch technology. We found that some people reported difficulty remembering to wear the smartband daily and the smartband was worn on average for 8.5 hours (SD = 4.9) daily. Adherence may be improved by using wearable technology that someone already owns and is familiar with, as compared to remembering to wear a smartband just for the study. Although the smartband is not currently FDA-regulated as a medical device, future clinical trials could provide supporting evidence for this approval.

In conclusion, these results provide initial support for the feasibility, acceptability, and potential efficacy of using smartband technology to provide real-time smoking monitoring for adults in an outpatient hospital tobacco treatment program. Findings show promise that an older outpatient population can use this innovative technology with high rates of adherence and can benefit from real-time tracking of smoking behavior. As the wearable market continues to grow, there is wide potential to test the use of this technology to support health and behavior change.

## Supporting information

S1 Data Data table. (XLS)

## References

[1] USDHHS. The health consequences of smoking—50 years of progress: a report of the Surgeon General. Atlanta (GA): US Department of Health and Human Services, Centers for Disease Control and Prevention, National Center for Chronic Disease Prevention and Health Promotion, Office on Smoking and Health; 2014. 17 p.

[2] Jamal A, Phillips E, Gentzke AS, Homa DM, Babb SD, King BA, et al. Current cigarette smoking among adults—United States, 2016. MMWR Morb Mortal Wkly Rep. 2018;67(2):53. 29346338 10.15585/mmwr.mm6702a1 PMC5772802

[3] Fiore MC, Jaen CR, Baker TB, Bailey WC, Benowitz NL, Curry SJ, et al. Treating tobacco use and dependence: 2008 update. Clinical Practice Guideline. Rockville (MD): U.S. Department of Health and Human Services, Public Health Service; 2008.

[4] Borland R, Partos TR, Yong H-H, Cummings KM, Hyland A. How much unsuccessful quitting activity is going on among adult smokers? Data from the International Tobacco Control Four Country cohort survey. Addiction. 2012;107(3):673–82. doi: 10.1111/j.1360-0443.2011.03685.x 21992709 PMC3909986

[5] CDC. Quitting smoking among adults—United States, 2000–2015. MMWR Morb Mortal Wkly Rep. 2017;65(52):1457–64. 28056007 10.15585/mmwr.mm6552a1

[6] Hughes JR, Keely J, Naud S. Shape of the relapse curve and long-term abstinence among untreated smokers. Addiction. 2004;99(1):29–38. doi: 10.1111/j.1360-0443.2004.00540.x 14678060

[7] Centers for Disease Control and Prevention. Quitting smoking among adults - United States, 2000-2015. MMWR Morb Mortal Wkly Rep [Internet]; 2017;65(52):1457–64. Available from: https://www.cdc.gov/mmwr/volumes/65/wr/mm6552a1.htm 28056007 10.15585/mmwr.mm6552a1

[8] Tiffany ST. A cognitive model of drug urges and drug-use behavior: role of automatic and nonautomatic processes. Psychol Rev. 1990;97(2):147–68. doi: 10.1037/0033-295x.97.2.147 2186423

[9] Orbell S, Verplanken B. The automatic component of habit in health behavior: habit as cue-contingent automaticity. Health Psychol. 2010;29(4):374–83. doi: 10.1037/a0019596 20658824

[10] Hunt WA, Matarazzo JD, Weiss SM, Gentry WD. Associative learning, habit, and health behavior. J Behav Med. 1979;2(2):111–24. doi: 10.1007/BF00846661 555484

[11] Piper ME, Piasecki TM, Federman EB, Bolt DM, Smith SS, Fiore MC, et al. A multiple motives approach to tobacco dependence: the Wisconsin Inventory of Smoking Dependence Motives (WISDM-68). J Consult Clin Psychol. 2004;72(2):139–54. doi: 10.1037/0022-006X.72.2.139 15065950

[12] Ajzen I. From intentions to actions: a theory of planned behavior. In: Action control. Springer; 1985. p. 11–39.

[13] Norman P, Conner M, Bell R. The theory of planned behavior and smoking cessation. Health Psychol. 1999;18(1):89–94. doi: 10.1037//0278-6133.18.1.89 9925050

[14] Ajzen I. Perceived behavioral control, self‐efficacy, locus of control, and the theory of planned behavior. J Appl Soc Psychol. 2002;32(4):665–83.

[15] Bandura A. Social cognitive theory of self-regulation. Organ Behav Hum Decis Process. 1991;50(2):248–87.

[16] Kanfer FH. Self-monitoring: methodological limitations and clinical applications. J Consult Clin Psychol. 1970;35:148–52.

[17] McFall RM. Effects of self-monitoring on normal smoking behavior. J Consult Clin Psychol. 1970;35(2):135–42. doi: 10.1037/h0030087 5474283

[18] McFall RM, Hammen CL. Motivation, structure, and self-monitoring: role of nonspecific factors in smoking reduction. J Consult Clin Psychol. 1971;37(1):80–6. doi: 10.1037/h0031279 5565631

[19] Shiffman S, Stone AA, Hufford MR. Ecological momentary assessment. Annu Rev Clin Psychol. 2008;4:1–32. doi: 10.1146/annurev.clinpsy.3.022806.091415 18509902

[20] Stone A, Shiffman S, Atienza A, Nebeling L. The science of real-time data capture: Self-reports in health research. Oxford University Press; 2007.

[21] Moghaddam NG, Ferguson E. Smoking, mood regulation, and personality: an event-sampling exploration of potential models and moderation. J Pers. 2007;75(3):451–78. doi: 10.1111/j.1467-6494.2007.00445.x 17489888

[22] Senyurek V, Imtiaz M, Belsare P, Tiffany S, Sazonov E. Cigarette smoking detection with an inertial sensor and a smart lighter. Sensors (Basel). 2019;19(3). 10.3390/s19030570 PMC6387353 30700056

[23] Imtiaz MH, Ramos-Garcia RI, Wattal S, Tiffany S, Sazonov E. Wearable sensors for monitoring of cigarette smoking in free-living: a systematic review. Sensors (Basel). 2019;19(21):4678. doi: 10.3390/s19214678 31661856 PMC6864810

[24] Skinner AL, Stone CJ, Doughty H, Munafò MR. StopWatch: the preliminary evaluation of a smartwatch-based system for passive detection of cigarette smoking. Nicotine Tob Res. 2019;21(2):257–61. doi: 10.1093/ntr/nty008 29373720 PMC6042639

[25] Cole CA, Anshari D, Lambert V, Thrasher JF, Valafar H. Detecting smoking events using accelerometer data collected via smartwatch technology: validation study. JMIR mHealth uHealth. 2017;5(12):e189. 10.2196/mhealth.9035 PMC5745355 29237580

[26] Parate A, Chiu M-C, Chadowitz C, Ganesan D, Kalogerakis E. RisQ: recognizing smoking gestures with inertial sensors on a wristband. MobiSys. 2014;2014:149–61. doi: 10.1145/2594368.2594379 26688835 PMC4682919

[27] Sazonov E, Lopez-Meyer P, Tiffany S. A wearable sensor system for monitoring cigarette smoking. J Stud Alcohol Drugs. 2013;74(6):956–64. doi: 10.15288/jsad.2013.74.956 24172124 PMC3817052

[28] Dar R. Effect of real-time monitoring and notification of smoking episodes on smoking reduction: a pilot study of a novel smoking cessation app. Nicotine Tob Res. 2018;20(12):1515–8. doi: 10.1093/ntr/ntx223 29126209

[29] Cole CA, Powers S, Tomko RL, Froeliger B, Valafar H. Quantification of smoking characteristics using smartwatch technology: pilot feasibility study of new technology. JMIR Form Res. 2021;5(2):e20464. doi: 10.2196/20464 33544083 PMC7895644

[30] Morriscey C, Shephard A, van Houdt A, Kerr D, Barrett SP. Using ‘Smart’ technology to aid in cigarette smoking cessation: examining an innovative way to monitor and improve quit attempt outcomes. J Smok Cessat. 2018;14(3):149–54. doi: 10.1017/jsc.2018.33

[31] Dar R. Effect of real-time monitoring and notification of smoking episodes on smoking reduction: a pilot study of a novel smoking cessation app. Nicotine Tob Res. 2018;20(12):1515–8. doi: 10.1093/ntr/ntx223 29126209

[32] Morriscey C, Shephard A, van Houdt A, Kerr D, Barrett SP. Using ‘Smart’ technology to aid in cigarette smoking cessation: examining an innovative way to monitor and improve quit attempt outcomes. J Smok Cessat. 2018;14(3):149–54. doi: 10.1017/jsc.2018.33

[33] Shadel WG, Edelen MO, Tucker JS, Stucky BD, Hansen M, Cai L. Development of the PROMIS nicotine dependence item banks. Nicotine Tob Res. 2014;16 Suppl 3(Suppl 3):S190-201. doi: 10.1093/ntr/ntu032 25118226 PMC4189402

[34] Biener L, Abrams DB. The Contemplation Ladder: validation of a measure of readiness to consider smoking cessation. Health Psychol. 1991;10(5):360–5. doi: 10.1037//0278-6133.10.5.360 1935872

[35] Sobell L, Sobell M. Alcohol consumption measures. In: Allen J, Columbus M, editors. Assessing alcohol problems: a guide for clinicians and researchers. Bethesda (MD): National Institute on Alcohol Abuse & Alcoholism; 1995. p. 55–73.

[36] Sobell L, Sobell M. Alcohol consumption measures. In: Wilson B, editors. Assessing alcohol problems: a guide for clinicians and researchers. Bethesda (MD): National Institute on Alcohol Abuse & Alcoholism; 2003. p. 75–99.

[37] Schober P, Vetter TR. Repeated measures designs and analysis of longitudinal data: if at first you do not succeed-try, try again. Anesth Analg. 2018;127(2):569–75. doi: 10.1213/ANE.0000000000003511 29905618 PMC6072386

[38] Johnson AL, Avila JC, Christensen L, Fahey MC, Jang J, Jarvis S. Smoking cessation treatment efficacy and impact on health outcomes among middle-aged and older adults: a scoping review. Nicotine Tob Res. 2025:ntaf122. doi: 10.1093/ntr/ntaf122 40476739

[39] Statistica. Number of connected wearable devices worldwide from 2019 to 2022; 2023.

[40] Pew Research Center. 21% of Americans say they use smart watches or fitness trackers; 2020. Available from: https://www.pewresearch.org/short-reads/2020/01/09/about-one-in-five-americans-use-a-smart-watch-or-fitness-tracker/ft_2020-01-09_fitnesstrackers_01a/

[41] Grand View Research. Wearable technology market size, share and trends analysis report by product 2025-2030; 2024. Available from: https://www.grandviewresearch.com/industry-analysis/wearable-technology-market

[42] Bashir U. Wearable device ownership in selected countries 2024; 2024. Available from: https://www.statista.com/forecasts/1101101/wearable-devices-ownership-in-selected-countries

[43] Rojewski AM, Baldassarri S, Cooperman NA, Gritz ER, Leone FT, Piper ME, et al. Exploring issues of comorbid conditions in people who smoke. Nicotine Tob Res. 2016;18(8):1684–96. doi: 10.1093/ntr/ntw016 26783291 PMC4941598

[44] Han B, Einstein EB, Compton WM. Patterns and characteristics of nicotine dependence among adults with cigarette use in the US, 2006-2019. JAMA Netw. 2023;6(6):e2319602-e. 10.1001/jamanetworkopen.2023.19602 PMC10290248 37351884

[45] Horvath M, Pittman B, O’Malley SS, Grutman A, Khan N, Gueorguieva R, et al. Smartband-based smoking detection and real-time brief mindfulness intervention: findings from a feasibility clinical trial. Ann Med. 2024;56(1):2352803. doi: 10.1080/07853890.2024.2352803 38823419 PMC11146247

[46] Schnall R, Liu J, Alvarez G, Porras T, Ganzhorn S, Boerner S, et al. A smoking cessation mobile app for persons living with HIV: preliminary efficacy and feasibility study. JMIR Form Res. 2022;6(8):e28626. doi: 10.2196/28626 35980739 PMC9437787

[47] Yang M-J, Sutton SK, Hernandez LM, Jones SR, Wetter DW, Kumar S, et al. A Just-In-Time Adaptive intervention (JITAI) for smoking cessation: feasibility and acceptability findings. Addict Behav. 2023;136:107467. doi: 10.1016/j.addbeh.2022.107467 36037610 PMC10246550

